# One-year reproductive effects of anti-GnRH and rabies vaccination in female pet cats

**DOI:** 10.1530/RAF-26-0102

**Published:** 2026-10-19

**Authors:** Shiri Novak, Boris Yakobson, Roni King, Lauren Jaebker, Douglas C Eckery, Tal Raz

**Affiliations:** ^1^ Koret School of Veterinary Medicine, The Robert H. Smith Faculty of Agricultural, Food & Environment, The Hebrew University of Jerusalem, Rehovot, Israel; ^2^ Kimron Veterinary Institute, Ministry of Agriculture, Rishon Lezion, Israel; ^3^ Nature & Parks Authority, Jerusalem, Israel; ^4^ USDA APHIS Wildlife Services, National Wildlife Research Center, Fort Collins, Colorado, USA; ^5^ Advanced Academic Programs, Krieger School of Arts and Sciences, Johns Hopkins University, Baltimore, Maryland, USA

**Keywords:** immunocontraception, anti-GnRH vaccine, gonacon, queens, felis catus, rabies, feline population management

## Abstract

**Abstract:**

Overpopulation of domestic cats poses risks to public health, biodiversity, and animal welfare. Surgical sterilization is widely used but has substantial limitations. Gonacon, an anti-GnRH vaccine, provides a nonsurgical alternative; however, data are lacking for owned queens living in natural habitats and for simultaneous vaccination against rabies. We evaluated the 1-year immunogenicity, safety, and efficacy of simultaneous Gonacon–rabies vaccination in pet queens and compared responses between indoor and outdoor cats. In a prospective field study, 36 intact queens (≥6 months) received Gonacon–rabies vaccination (Gonacon–Rabies, *n* = 30) or ovariohysterectomy with rabies vaccination (Ovx–Rabies, *n* = 6). Queens remained under their owners’ care and were monitored at 0, 3, 6, and 12 months. Twenty-five queens completed the follow-up (22 Gonacon–Rabies, 3 Ovx–Rabies). Injection-site nodules were observed only following Gonacon vaccination (13.6 vs 0%; *P* = 0.0564). Anti-GnRH seropositivity was detected in 68.2, 31.8, and 27.3% of Gonacon–Rabies queens at 3, 6, and 12 months, respectively. Among responders (*n* = 15), 53.3% were positive only at 3 months, 6.7% remained positive up to 6 months, and 40% remained positive throughout the study. All Gonacon-vaccinated nonresponders were outdoor queens (outdoor: 7/13; 53.8%; indoor: 0/9; *P* = 0.0167). The duration of anti-GnRH immunity was shorter in outdoor queens (2.1 ± 0.9 vs 8.3 ± 1.5 months; *P* = 0.0005). Reproductive events (estrus or pregnancy) occurred in 76.9% of outdoor vs 11.1% of indoor Gonacon–Rabies queens (*P* = 0.0075). AMH concentrations were inversely correlated with anti-GnRH levels (*r* = −0.53; *P* < 0.0001). Rabies antibodies remained protective (≥0.5 IU/mL) but were lower in outdoor vs indoor queens (*P* = 0.0490). Owner satisfaction was higher for Gonacon–Rabies indoor queens and those without reproductive events. Simultaneous Gonacon–rabies vaccination was safe and partially effective over 1 year, with better responsiveness in indoor queens.

**Lay summary:**

Domestic cats are highly prolific, contributing to overpopulation that negatively affects animal welfare, wildlife biodiversity, and public health. Although surgical sterilization is widely used to prevent reproduction in cats, it has important practical, economic, and ethical limitations. We studied the safety, immune response, and reproductive effects of a contraceptive anti-GnRH vaccine (Gonacon) administered concurrently with a rabies vaccine in female pet cats living under normal home conditions. The combined vaccination was generally safe, and most queens maintained protective immunity against rabies. However, the contraceptive effects varied considerably among animals. Indoor queens developed stronger, longer-lasting immune responses and experienced fewer signs of estrus and fewer pregnancies than outdoor queens. In contrast, many outdoor queens showed weaker and shorter-lasting immune responses. These findings suggest that combined vaccination may provide a practical nonsurgical approach to controlling reproduction in some pet cats, but living conditions can strongly influence its effectiveness.

## Introduction

Overpopulation of domestic cats poses a significant global challenge in both urban and rural regions (Gunther *et al.* 2015, 2022, Ma *et al.* 2023). The global domestic cat population is estimated at several hundred million individuals, many of which are owned as pets, and household ownership of multiple cats is common (Kennedy *et al.* 2020). Owned or pet cats typically fall into one of three habitat categories: indoor cats, confined solely within the household; indoor/outdoor cats, living indoors with access to outdoor environments; and outdoor cats, living predominantly outdoors but still receiving varying degrees of care from their owners, such as food, shelter, and medical treatment (Ma *et al.* 2023). The uncontrolled breeding of owned outdoor cats, coupled with the proliferation of stray and abandoned cats, has led to the emergence of free-roaming cat populations across numerous regions worldwide (Gunther *et al.* 2020, Kennedy *et al.* 2020, Ma *et al.* 2023).

Overpopulation of free-roaming cats is associated with substantial public health risks due to the transmission of zoonotic diseases to humans, such as rabies, toxoplasmosis, cat scratch disease, leptospirosis, Q fever, and toxocariasis (Gerhold & Jessup 2013, Roebling *et al.* 2014). Cats may also mediate the transmission of diseases to livestock and wild animals (Wareth *et al.* 2017, Trouwborst *et al.* 2020). In addition, they may attack humans, be involved in traffic accidents, and cause nuisances, such as the presence of cat carcasses, noise and dirt, and invasion into private or business grounds (Gunther *et al.* 2015, Crawford *et al.* 2019). Moreover, free-roaming and feral cats are non-native predators that commonly kill birds, reptiles, amphibians, small mammals, and fish, with many of these prey species being on the verge of extinction (Medina *et al.* 2011, Loss *et al.* 2022). Due to these significant ecological disruptions and wildlife depletion, the domestic cat has been included among the world’s top 100 worst non-native invasive species (Lowe *et al.* 2000, Loss *et al.* 2022). In addition, overpopulation of free-roaming cats is associated with high prevalences of malnutrition, disease susceptibility, and injuries among cat populations, leading to adverse effects on their welfare and health (Gunther *et al.* 2015, 2018, Ma *et al.* 2023).

Rabies, a lethal zoonotic viral disease affecting both humans and animals, is endemic in many countries (Boutelle *et al.* 2025). According to World Health Organization reports published between 2020 and 2024, rabies causes an estimated 59,000–60,000 human deaths annually worldwide, with the vast majority resulting from dog-mediated transmission. Nevertheless, in many countries, including the United States, cats are the most frequently reported rabid companion animal (Gerhold & Jessup 2013, Ma *et al.* 2024, Boutelle *et al.* 2025). Consequently, in the last few decades, efforts have been made to control the overpopulation of free-roaming cats and prevent rabies (Roebling *et al.* 2014). However, the influence of different habitats on rabies vaccine immunogenicity and responsiveness in domestic cats remains unclear.

Currently, surgical sterilization is the primary method for preventing fertility in owned cats and for managing free-roaming cat populations, typically through trap–neuter–vaccinate (for rabies)-release (TNVR) programs (Roebling *et al.* 2014, Murray *et al.* 2015, Gunther *et al.* 2022). Nevertheless, the surgical approach has clear limitations. The need for anesthetic materials, specialized equipment, the expertise of skilled veterinarians, and, in the case of free-roaming cats, the additional costs of trapping and transporting them to a proper facility collectively contribute to its substantial financial and practical burdens (Lohr *et al.* 2013, Benka *et al.* 2022, Gunther *et al.* 2022). Moreover, surgical procedures cause considerable stress, pain, and discomfort to the animals involved (Pereira *et al.* 2018, Crawford *et al.* 2019). The invasiveness of the procedures, along with the subsequent recovery period and the unfamiliar surroundings of veterinary clinics, contribute to the distress experienced by cats, which is a concern not only from an ethical standpoint but also due to adverse health effects (Adin 2011, Pereira *et al.* 2018, Klintip *et al.* 2022). Furthermore, surgical sterilization procedures in cats can lead to intraoperative and postoperative sequelae, such as anesthetic complications, bleeding, surgical-site infections, wound dehiscence, adverse reactions to sutures, seroma or hematoma formation, and urinary tract issues (Adin 2011, Pereira *et al.* 2018). Hence, despite its advantage of providing a permanent resolution for the individual animal, the intrinsic constraints related to cost, stress, health implications, and logistical challenges have hindered the scalability and efficacy of the surgical approach. Practically, the surgical approach has failed to mitigate the extensive reproduction of free-roaming cat populations (Root Kustritz 2012, Roebling *et al.* 2014, Benka & Levy 2015, Crawford *et al.* 2019). In addition, many cat owners are reluctant to subject their pets to surgical interventions due to these concerns or ethical and religious convictions (Root Kustritz 2012, Murray *et al.* 2015). Nevertheless, commercial products for mass non-surgical sterilization of owned and free-roaming cats are currently unavailable despite extensive research efforts (Kutzler & Wood 2006, Benka & Levy 2015).

Gonacon is an anti-GnRH vaccine developed by the National Wildlife Research Center, USDA APHIS Wildlife Services, USA, that aims to suppress reproduction by generating GnRH-neutralizing antibodies (Miller *et al.* 2008). Previous studies examining its effects on cats have mostly involved animals in non-natural settings (Levy *et al.* 2011, Vansandt *et al.* 2017, Fischer *et al.* 2018), such as laboratory or research colony cats, that differ significantly from feral and outdoor cats exposed to a variety of environmental stressors and health challenges (Slater 2007, Gunther *et al.* 2015, Crawford *et al.* 2019). Notably, there is limited information regarding the immunogenicity of Gonacon and rabies vaccines when administered concurrently to the same cat, which hampers evidence-based decision-making for programs that rely on coordinated contraceptive and rabies control strategies. Furthermore, data on Gonacon vaccination, either alone or in combination with rabies vaccination, in owner-owned pet cats living in their natural environments (indoors or outdoors) have been very limited. Recently, however, we reported the 1-year safety, immunogenicity, and reproductive effects of combined anti-GnRH and rabies vaccination in owner-owned male cats (Novak *et al.* 2026). Information regarding the safety, immunogenicity, and efficacy of combined Gonacon–rabies vaccination in owner-owned female cats (queens) living under natural conditions, however, remains scarce. To address this knowledge gap, we also conducted a parallel 1-year study in owner-owned female pet cats, which we report herein.

The primary objective of the current study was to evaluate the safety, immunogenicity, and efficacy of simultaneous Gonacon and rabies vaccination over a 1-year period in owner-owned queens living in their natural environments and to compare responses between indoor and outdoor cats. Our main hypotheses were that: i) Gonacon–rabies vaccination would be safe and would not result in adverse effects; ii) antibody responses against GnRH and rabies would be maintained throughout the study period and would not differ between indoor and outdoor queens; iii) rabies antibody responses would not be negatively affected by simultaneous vaccination and would remain protective (≥0.5 IU/mL) throughout the study period; and iv) anti-GnRH seropositivity would be associated with suppression of reproductive activity.

## Materials and methods

### Animals and study design

The study was conducted in accordance with the Hebrew University Institutional Animal Care and Use Committee (IACUC) ethical approval (MD-15-1411-2). Cat owners in the central area of Israel were invited to enroll their queens in this study via email to veterinary clinics in the region, to Koret School of Veterinary Medicine staff and students, and through online advertisements (e.g. animal-related Facebook groups and websites). Owners of queens who contacted the research team received the relevant information, and the animals were enrolled voluntarily by their owners, without compensation. Informed consent was obtained and signed by all owners prior to their queen’s enrollment in the study. Queens were enrolled and received their assigned treatment when they became available for participation; consequently, individual queens entered the study at different times throughout the year. A licensed veterinarian performed all animal procedures in the study (S N). The inclusion criteria were intact queens aged 6 months or older, with no abnormalities detected during veterinary physical examination. Queens included in the study were considered rabies vaccine naïve at enrollment based on owner-reported vaccination history and the absence of detectable rabies antibody concentrations prior to vaccination.

A total of 36 queens were initially recruited into this field-based study and allocated to one of two groups. At study initiation, queens in the Gonacon–Rabies group (*n* = 30) were vaccinated with both anti-GnRH (Gonacon) and rabies vaccines, whereas in the Ovx–Rabies group, queens (*n* = 6) underwent surgical ovariohysterectomy and were vaccinated against rabies. Throughout the 1-year study, queens in both groups remained in their natural habitat, under the care and possession of their owners.

### Treatments, sampling, and monitoring

Queens were examined and sampled by the research team four times throughout the study: at study initiation (time 0, when the initial treatments were performed; i.e. vaccinations and ovariohysterectomy), and at 3, 6, and 12 months after vaccination. Queens were sedated for veterinary physical examination, vaccinations, and blood sample collections by combining medetomidine (0.02–0.04 mg/kg; Orion Pharma, Finland), butorphanol (0.2–0.4 mg/kg; intramuscular injection; Richter Pharma AG, Austria), and midazolam (0.1–0.25 mg/kg intramuscular injection; Rafa Laboratories, Israel). Medetomidine’s sedative effects were counteracted at the end of the procedure by administering atipamezole subcutaneously (0.05–0.10 mg/kg; Orion Pharma, Finland) to facilitate recovery. The queens were released back to their homes and the care of their owners only after they had fully recovered from the sedation and were bright, alert, and responsive.

At study initiation, each queen was vaccinated against rabies by subcutaneous injection of a licensed rabies vaccine (Rabisin; Merial, France) in the left thigh. Simultaneously, Gonacon immunization was delivered intramuscularly using vaccine material provided by USDA-APHIS NWRC (Fort Collins, CO, USA). A 500 μL dose was injected into the right quadriceps, as previously described (Novak *et al.* 2021, 2026). The fur over each injection site was clipped, and the skin was prepared with 70% isopropyl alcohol before administration.

At each examination, a complete physical examination was performed by a certified veterinarian, including evaluation of body condition on a 5-point scale (Baldwin *et al.* 2010) and examinations of the injection sites. Blood samples were collected by jugular venipuncture into plain tubes without anticoagulant and into potassium-EDTA tubes. Blood in plain tubes was allowed to clot, centrifuged, and the serum samples were stored at −80°C until analysis at study completion; these analyses included anti-rabies and anti-GnRH antibody levels, serology for FeLV and FIV, and analysis of serum anti-Müllerian hormone (AMH) concentration. Blood samples in potassium-EDTA tubes were used to measure packed cell volume (PCV), using a hematocrit tube sealed with clay that was centrifuged for approximately 3 min; plasma total solids (TS) were measured by placing the plasma directly onto a refractometer (Clinical Refractometer RHC-200 ATC, Cmall, Medent, Israel).

Every 3 months, either during scheduled examinations or by telephone follow-up, owners were asked to complete a questionnaire regarding their queen’s housing conditions (i.e. indoor or outdoor), general health status, and reproductive behavior. In addition, throughout the study period, owners were instructed to report to the research team in real time any behavioral or health abnormalities, signs of estrus, or reproductive-related events, including mating, pregnancy, or parturition.

### FIV/FeLV serology

To further assess overall health status, serum collected at the midpoint of the study (6 months post-vaccination) was tested for feline immunodeficiency virus (FIV) antibodies and feline leukemia virus (FeLV) p27 antigen. Analyses were performed using a commercial FIV/FeLV test kit (SNAP Combo Plus, IDEXX, USA) according to the manufacturer’s instructions. The queens’ FeLV vaccination histories were unknown; however, such vaccination is not expected to interfere with FeLV antigen detection using the SNAP Combo Plus assay (Little *et al.* 2020).

### Detection of anti-GnRH antibodies

Serum samples collected at 0, 3, 6, and 12 months were stored frozen and shipped on study completion to the USDA-APHIS National Wildlife Research Center (Fort Collins, CO, USA) for analysis of anti-GnRH antibodies by enzyme-linked immunosorbent assay (ELISA), following previously published procedures (Levy *et al.* 2011, Novak *et al.* 2021, 2026). Serum samples were diluted 1:8,000 in phosphate-buffered saline (PBS). All samples were analyzed in duplicate, and antibody responses were quantified using optical density (OD) measurements. A positive control sample was included in duplicate on each assay plate to monitor assay performance. The threshold for anti-GnRH seropositivity was established using the pre-vaccination samples from all queens and was defined as the mean OD value plus three standard deviations. Samples with OD values exceeding this threshold were classified as seropositive, whereas those below the threshold were considered seronegative. To minimize analytical variation, all samples were analyzed by the same operator, who was blinded to sample identity, treatment groups, and housing status. In addition, all samples from an individual queen were analyzed within the same assay run. The intra-assay and interassay coefficients of variation were 12.3 and 9.5%, respectively.

### Detection of anti-rabies antibodies

Serum samples collected at 0, 3, 6, and 12 months were analyzed for rabies virus-neutralizing antibodies using the rapid fluorescent focus inhibition test (RFFIT), performed by the WOAH-accredited Rabies Reference Laboratory at the Kimron Veterinary Institute (Veterinary Services and Animal Health, Ministry of Agriculture, Israel), as previously described (Yakobson *et al.* 2015, Novak *et al.* 2021, 2026). RFFIT is considered the reference method for the measurement of rabies virus-neutralizing antibodies and has demonstrated high diagnostic performance (i.e. 100% sensitivity and 89% specificity); it is therefore considered the gold standard by the World Health Organization (Bahloul *et al.* 2005, Yakobson *et al.* 2015). Briefly, serial serum dilutions were incubated with rabies virus in the presence of positive and negative controls, followed by the addition of mouse neuroblastoma cells (CDC, Atlanta, GA, USA). After incubation, the cells were fixed, stained with a fluorescein isothiocyanate (FITC)-conjugated anti-rabies antibody (Fujirebio, USA), and examined by fluorescence microscopy (Olympus BX40; excitation, 470 nm; ×200 magnification). Rabies virus-neutralizing antibody concentrations were calculated and expressed as international units per milliliter (IU/mL). Antibody concentrations ≥0.5 IU/mL were considered indicative of adequate serological protection (Yakobson *et al.* 2015).

### Serum anti-Müllerian hormone analysis

Serum AMH concentrations were quantified in all collected samples using a commercial ELISA kit (AMH Gen II; Beckman Coulter Inc., USA), according to the manufacturer’s protocol and previously published methods validated for feline samples (Axner & Strom Holst 2015, Novak *et al.* 2021). Briefly, 300 μL of AMH Gen II assay buffer was combined with 60 μL of calibrator, control, or serum sample, and 120 μL of this mixture was dispensed in duplicate into the assay plate. After an incubation and subsequent washing, 100 μL biotin-labeled anti-AMH antibody was added. Plates were incubated and washed again before adding 100 μL streptavidin–enzyme conjugate. Following a third incubation and wash cycle, 100 μL TMB substrate solution was introduced, and the reaction was terminated by adding 100 μL supplied stop solution. Absorbance was measured at 450 nm with reference correction at 620 nm using a SpectraMax Paradigm multi-mode microplate reader (SpectraMax Paradigm Multi-Mode Detection Platform, Molecular Devices, Austria). The assay demonstrated inter- and intra-assay coefficients of variation of 8.2 and 2.4%, respectively.

### Owner satisfaction

At the completion of the 1-year follow-up period, owners of queens in the Gonacon–Rabies group were asked to complete a brief questionnaire evaluating their overall satisfaction with the Gonacon–rabies vaccination strategy and their likelihood of recommending it to other owners of intact female cats. The questionnaire included two items: i) whether the owner was satisfied with the effectiveness of Gonacon–rabies vaccination in suppressing reproductive-related signs in their queen; ii) whether they would recommend this vaccination approach to other cat owners. Throughout the study, owners were blinded to the hormonal and immunological findings obtained from their cats.

### Statistical analysis

Statistical analyses were performed using Statistix 8 software (Analytical Software, USA); plots were produced using Prism 5.01 (GraphPad Software, USA). Data were compared between the Gonacon–Rabies and Ovx–Rabies groups, and between the indoor and outdoor cat habitat groups. Continuous data were analyzed by Repeated Measures ANOVA, to evaluate the effects of Group (between-subject factor), Time (within-subject factor), and Group-by-Time interactions, followed by Tukey HSD All-Pairwise Comparisons Test. The month and season of vaccination or sampling had no impact on the statistical outcomes and were therefore excluded from the final analytic model, consistent with previous reports indicating that domestic cats in warm and temperate countries, including Israel, can breed throughout most of the year (Dards 1983, Gunther *et al.* 2015). When applicable, two groups were compared using the Wilcoxon Rank Sum Test. The correlation between serum AMH concentrations and anti-GnRH antibody levels (OD) was evaluated by the Pearson Correlation Test. Serum AMH concentrations in queens with negative vs positive anti-GnRH antibody levels at different time points post-vaccination were analyzed using a General AOV/AOCV. Fisher’s Exact Test or the Chi-square Test was used to compare proportions. Differences were considered significant at *P* < 0.05. Unless otherwise noted, results are presented as mean ± SEM.

## Results

Of the 36 queens initially enrolled, 25 completed the 1-year follow-up, including 22 from the Gonacon–Rabies group (9 indoor, 13 outdoor) and 3 from the Ovx–Rabies group. Treatment initiation was distributed throughout the year. Among the 22 Gonacon–Rabies queens that completed follow-up, 3 (13.6%) were treated in fall, 8 (36.4%) in winter, 3 (13.6%) in spring, and 8 (36.4%) in summer. Of the three Ovx–Rabies queens, two were treated in summer and one in winter. The loss to follow-up of 11 queens corresponds to a 30.6% dropout rate. Reasons for loss to follow-up included queens that were difficult to capture for repeated testing beyond the initial examination (*n* = 5), were hit by a car and died (*n* = 2), disappeared (*n* = 1), or changed ownership (*n* = 1). In addition, the owners of two queens discontinued cooperation following the initial vaccination. Interestingly, 10 of these 11 follow-up losses were outdoor queens, indicating a clear tendency for a higher risk for follow-up loss among outdoor cats compared to indoor cats (indoor: 1/10, 10% vs outdoor: 10/26, 38.5%; odds ratio = 5.6; *P* = 0.0968).

### Physical examinations, PCV/TS, and FeLV/FIV analyses

During the study, none of the queens exhibited swelling, signs of inflammation, or pain at the rabies vaccine injection sites. However, three queens developed localized masses at the Gonacon vaccine injection site, first identified during scheduled examinations at 3, 6, and 12 months after vaccination, respectively. The mass detected at 3 months was relatively soft and measured approximately 3 × 2 cm; it subsequently became firm and decreased in size to approximately 2 × 1 cm by 12 months. The mass first detected at 6 months was firm and oval, measuring approximately 3.7 × 2 cm, and gradually decreased in size during follow-up. The mass first detected at 12 months was firm and measured approximately 2.5 × 2 cm. The corresponding statistical analysis indicated a tendency toward a higher risk of injection–site reaction following Gonacon vaccination compared with rabies vaccination (Gonacon: 3/22, 13.6%, vs Rabies: 0/25, 0%; *P* = 0.0564).

All queens were in good physical condition. Mean body condition score (BCS) did not differ between the Gonacon–Rabies and Ovx–Rabies groups, but overall, it tended to improve over the study period (Supplementary Fig. 1A (see the section on Supplementary materials given at the end of the article), Time factor, *P* = 0.0633); BCS of individual cats stayed the same or improved by up to one point in a 5-point BCS scale (Baldwin *et al.* 2010). Upon examination, vital signs (TPR: temperature, pulse, and respiratory rate) remained within normal limits, with no significant differences between the groups or over time (Supplementary Fig. 1B, C, D). The values of PCV and TS were within normal limits, with no significant differences between the Gonacon–Rabies and Ovx–Rabies groups or over time (Supplementary Fig. 1E and F). In the Gonacon–Rabies group, vital signs (TPR) and TS did not differ between indoor and outdoor queens; however, PCV was lower overall in outdoor cats (*P* = 0.0488). All queens in the study were negative for FeLV/FIV.

### GnRH antibodies

As expected, following vaccination, the optical density (OD) readings of anti-GnRH antibodies were higher in the Gonacon–Rabies group as compared to the Ovx–Rabies group (Fig. 1A, *P* = 0.0235). Overall, anti-GnRH seropositivity was detected in 68.2% (15/22) of Gonacon-vaccinated queens, indicating a non-response rate of 31.8%. Positive anti-GnRH antibody levels were detected in 0, 68.2, 31.8, and 27.3% of the Gonacon-vaccinated queens at 0, 3, 6, and 12 months after vaccination, respectively, indicating a significant increase in anti-GnRH antibodies after vaccination, followed by a reduction over time (Fig. 1A and B). Among the queens that responded positively to Gonacon (*n* = 15), 40% (6/15) remained positive at all examinations (3, 6, and 12 months); 6.7% (1/15) were positive at 3 and 6 months but not at 12 months; and 53.3% (8/15) were positive only at the 3-month examination.

**Figure 1:**
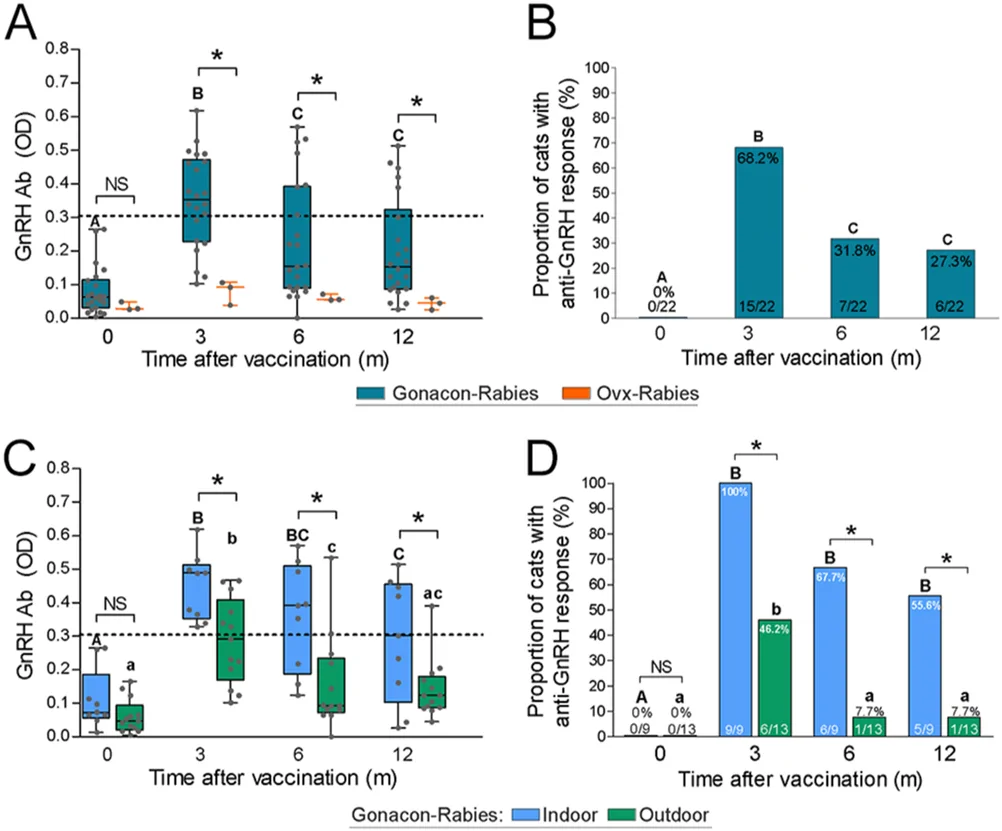
Serum anti-GnRH antibody levels in the Gonacon–Rabies and Ovx–Rabies groups and in indoor vs outdoor Gonacon-vaccinated queens. Queens in the Gonacon–Rabies group received simultaneous Gonacon and rabies vaccination, whereas queens in the Ovx–Rabies group underwent ovariohysterectomy and rabies vaccination. Measurements were obtained before treatment and at 3, 6, and 12 months thereafter. In panels A and C, boxplots show the median values (central line), interquartile range (box), and minimum and maximum values (whiskers), with individual observations overlaid (each dot represents a single sample). The horizontal dashed line indicates the anti-GnRH seropositivity cutoff. Data were analyzed using repeated-measures ANOVA with group or habitat, time, and their interaction effects, followed by Tukey HSD pairwise comparisons. Panels B and D show the proportions of queens classified as anti-GnRH seropositive after Gonacon vaccination. (A) Anti-GnRH antibody optical density (OD) values in Gonacon–Rabies and Ovx–Rabies queens (group *P* = 0.0235, time *P* = 0.0023, group × time *P* = 0.0564). (B) Proportion of Gonacon–Rabies queens seropositive for anti-GnRH antibodies over time. (C) Anti-GnRH antibody OD values in indoor and outdoor Gonacon–Rabies queens (habitat *P* = 0.0033, time *P* < 0.0001, habitat × time *P* = 0.1159). (D) Proportions of indoor and outdoor Gonacon–Rabies queens seropositive for anti-GnRH antibodies over time. Different letters (^A,B,C^ or ^a,b,c^) indicate significant time effects within a group. *P < 0.05; NS, not significant.

Interestingly, all Gonacon non-responder queens were outdoor cats (non-responder rate – indoor: 0%, 0/9; outdoor: 53.8%, 7/13; *P* = 0.0167). Moreover, GnRH antibody levels (OD) were significantly lower in outdoor vs indoor queens (Fig. 1C), as well as the proportions of queens with positive anti-GnRH response at the different time points after Gonacon vaccination (Fig. 1D). Accordingly, despite the variability among queens, the mean duration of anti-GnRH seropositivity was longer in indoor as compared to outdoor queens (indoor: 8.3 ± 1.5 months vs outdoor: 2.1 ± 0.9 months; *P* = 0.0005). Overall, as shown for the rabies vaccine, the immunogenicity of the Gonacon vaccine was lower in outdoor queens compared to indoor queens.

### Rabies antibodies

All queens produced antibodies against rabies following vaccination, with antibody levels maintained above 0.5 IU/mL throughout the study, a level that is considered protective (Mansfield *et al.* 2004, Shiraishi *et al.* 2014). There were no significant differences in the anti-rabies antibodies between the Gonacon–Rabies and Ovx–Rabies groups (Fig. 2A, *P* = 0.4428). Interestingly, however, rabies antibody concentrations were lower in outdoor queens than in indoor queens (Fig. 2B, *P* = 0.0490).

**Figure 2:**
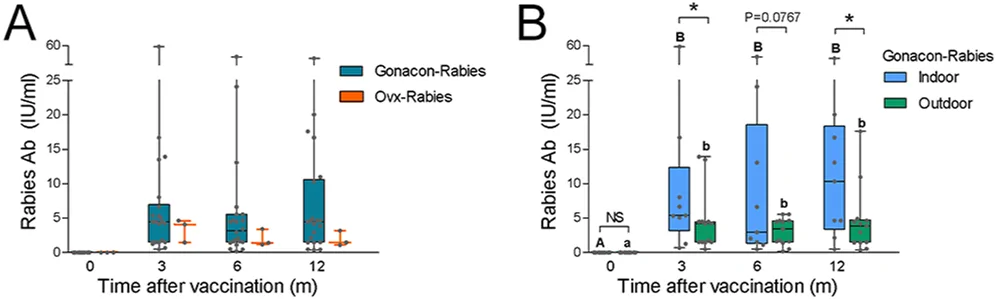
Serum rabies virus-neutralizing antibody concentrations in queens receiving Gonacon–Rabies vaccination or Ovx–Rabies treatment and in indoor and outdoor queens. Queens in the Gonacon–Rabies group received simultaneous Gonacon and rabies vaccination, whereas queens in the Ovx–Rabies group underwent ovariohysterectomy and rabies vaccination. Antibody concentrations were measured before treatment and at 3, 6, and 12 months thereafter. Boxplots show the median (central line), interquartile range (box), and minimum and maximum values (whiskers), with individual observations overlaid (each dot represents a value from a single sample). Data were analyzed using repeated-measures ANOVA to assess the effects of group or habitat (between-subject factor), time (within-subject factor), and the group × time or habitat × time interaction, followed by Tukey HSD pairwise comparisons when applicable. (A) Gonacon–Rabies vs Ovx–Rabies (group *P* = 0.4428, time *P* = 0.1182, group × time *P* = 0.6831). (B) Indoor vs outdoor queens (habitat *P* = 0.0490, time *P* < 0.0001, habitat × time *P* = 0.0301). Different letters (either ^A,B,C^, or ^a,b,c^) indicate significant time effects within a group. *P < 0.05; NS, not significant.

### Anti-Müllerian hormone serum concentrations

In the Ovx–Rabies group, serum AMH concentrations reduced significantly from 7.1 ± 1.2 ng/mL before ovariohysterectomy to nondetectable values at 3, 6, and 12 months later (Fig. 3A). In the Gonacon–Rabies group, mean AMH concentrations reduced significantly at 3 months after vaccination as compared to before vaccination, but did not differ at 6 and 12 months after vaccination (Fig. 3A). AMH concentrations did not differ between outdoor vs indoor queens vaccinated with Gonacon (Fig. 3B), but there was a tendency for lower AMH concentration at 3 months after vaccination in indoor vs outdoor queens (*P* = 0.0727). Interestingly, in a dichotomous analysis, serum samples with positive anti-GnRH response had lower AMH concentrations than samples with negative response (*P* < 0.0001; Fig. 3D). Furthermore, the relationship between anti-GnRH antibodies (OD) and AMH was found to be negatively correlated, with a Pearson correlation coefficient of −0.5324 (*P* < 0.0001; Fig. 3C). As the levels of anti-GnRH antibodies increased, particularly above the cutoff, the level of AMH decreased; however, when the anti-GnRH antibodies decreased, the AMH concentration increased. Moreover, an analysis of factors affecting AMH concentrations at different time points after vaccination revealed that the status of the anti-GnRH antibody response (positive or negative) at the time of sampling significantly influenced AMH concentrations (Fig. 3E).

**Figure 3:**
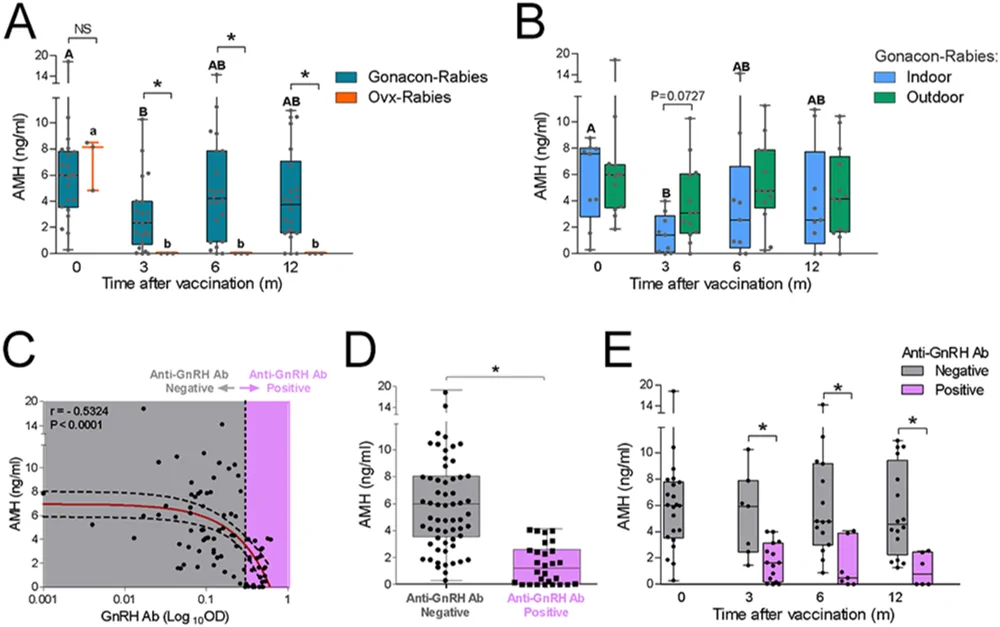
Serum AMH concentrations in the Gonacon–Rabies and Ovx–Rabies groups, in indoor and outdoor Gonacon-vaccinated queens, and their associations with anti-GnRH antibodies. Queens in the Gonacon–Rabies group received simultaneous Gonacon and rabies vaccination, whereas queens in the Ovx–Rabies group underwent ovariohysterectomy and rabies vaccination. Data were collected before vaccination and at 3, 6, and 12 months thereafter. All panels except C show box-and-whisker plots with overlaid scatter points. Boxplots display the median (central line), interquartile range (box), and minimum and maximum values (whiskers); each dot represents a single sample. (A) Serum AMH concentrations in the Gonacon–Rabies and Ovx–Rabies groups (group *P* = 0.0627, time *P* = 0.0004, group × time *P* = 0.0918; repeated-measures ANOVA). (B) Serum AMH concentrations in indoor and outdoor queens in the Gonacon–Rabies group (habitat *P* = 0.2378, time *P* = 0.0084, habitat × time *P* = 0.7914; repeated-measures ANOVA). (C) Correlation between serum AMH concentrations and anti-GnRH antibody (OD) levels (*r* = −0.5324, *P* < 0.0001; Pearson correlation test). The vertical dotted line indicates the anti-GnRH seropositivity cutoff; samples to the right (pink area) were classified as seropositive, and those to the left (gray area) as seronegative. (D) Serum AMH concentrations in samples classified as anti-GnRH seronegative or seropositive in the Gonacon–Rabies group (*P* < 0.0001; Wilcoxon rank-sum test). (E) Serum AMH concentrations according to anti-GnRH serostatus (seropositive vs seronegative) at different postvaccination time points (anti-GnRH serostatus *P* < 0.0001, time *P* = 0.7246, habitat *P* = 0.2500; general AOV/AOCV analysis). Different letters (^A,B,C^, or ^a,b,c^) indicate significant differences over time within a group. *P < 0.05; NS, not significant.

### Reproductive events

As noted, owners were asked to report signs of estrus and reproductive events such as mating, pregnancy, or parturition. As expected, queens in the Ovx–Rabies group had no reproductive events (Fig. 4A). However, in the Gonacon–Rabies group, 50% (11/22) of the queens had no reported reproductive events and appeared to remain reproductively suppressed throughout the 1-year monitoring period. Of the remaining 11 queens, 10 were reported as pregnant or having delivered kittens, and one showed signs of estrus. No reproductive events were reported during the first 6 months after vaccination; all first reported reproductive events occurred between 6 and 12 months after Gonacon vaccination (Fig. 4). Interestingly, the vast majority (10/11) occurred in outdoor queens (Fig. 4B). Overall, 76.9% (10/13) of the outdoor queens vaccinated with Gonacon were reported as pregnant or having delivered kittens during the study, while among the indoor queens, only one (1/9, 11.1%) showed signs of estrus (Fisher Exact Test, *P* = 0.0075). Reproductive events were associated with non-responders, reduced anti-GnRH antibody levels, and re-elevation of serum AMH concentrations, as reported above.

**Figure 4:**
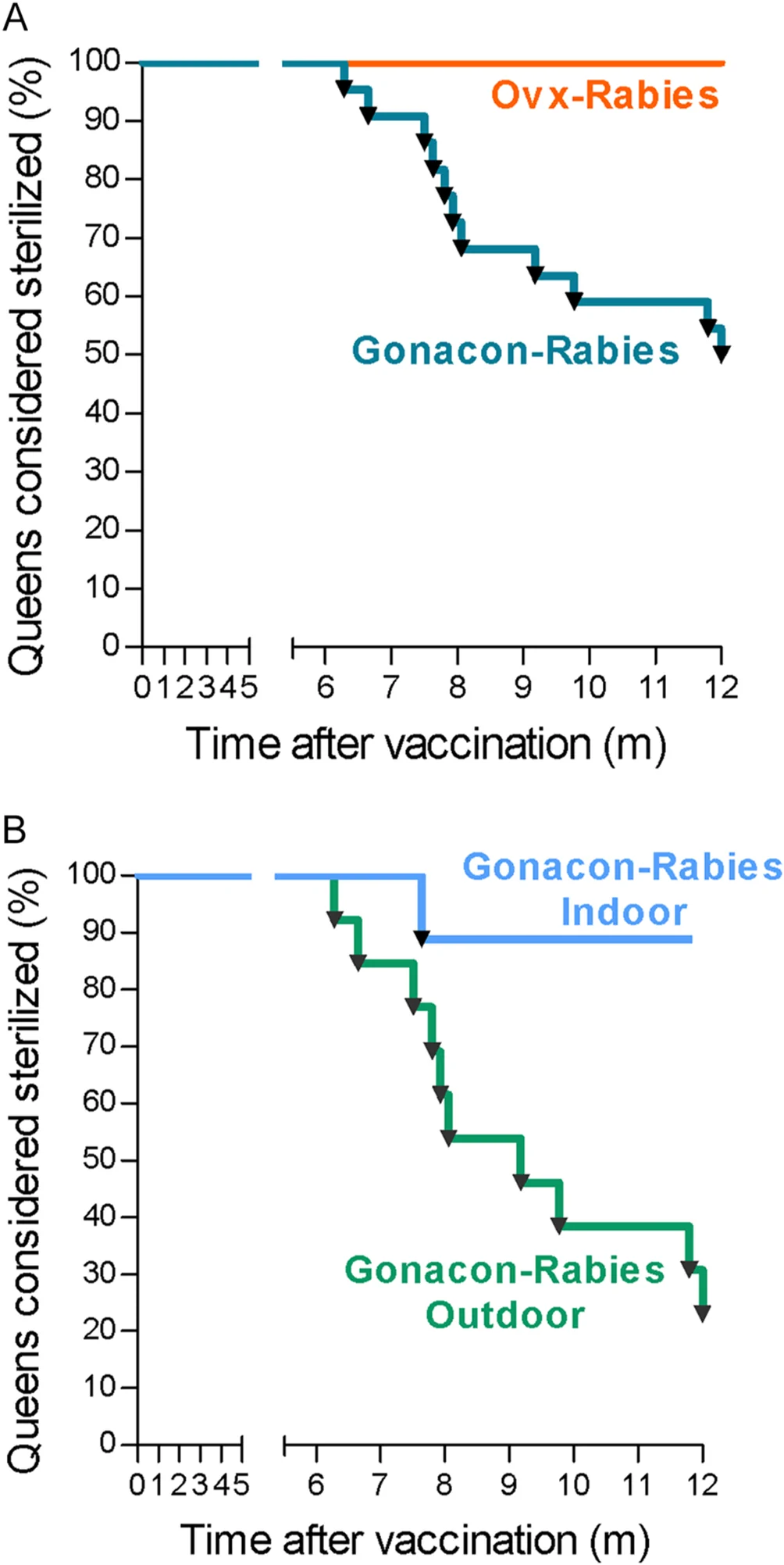
Reported reproductive events in the Gonacon–Rabies and Ovx–Rabies groups and in indoor and outdoor Gonacon-vaccinated queens. Queens in the Gonacon–Rabies group received simultaneous Gonacon and rabies vaccination, whereas queens in the Ovx–Rabies group underwent ovariohysterectomy and rabies vaccination. Signs of estrus and reproductive events (e.g. mating, pregnancy, and parturition) were documented through owner reports and routine examinations. For each queen, the time to the first reported reproductive event was used in the analysis. Among the 11 Gonacon–Rabies queens with a reported event, 10 were first reported as pregnant or as having delivered kittens, and one was reported with signs of estrus. (A) Kaplan–Meier analysis of the time to the first reported reproductive event in Gonacon–Rabies and Ovx–Rabies queens (log-rank Mantel–Cox test: *P* = 0.1016; Gehan–Breslow–Wilcoxon test: *P* = 0.1077). (B) Kaplan–Meier analysis of the time to the first reported reproductive event in indoor and outdoor Gonacon–Rabies queens (log-rank Mantel–Cox test: *P* = 0.0053; Gehan–Breslow–Wilcoxon test: *P* = 0.0101).

### Owner satisfaction

At the conclusion of the study, 59% (13/22) of owners expressed satisfaction with the Gonacon–rabies vaccination approach and indicated they would recommend it to other queen owners. On average, queens of owners who expressed positive satisfaction had a longer duration of positive anti-GnRH antibody levels than queens of owners who were not satisfied with it (6.7 ± 1.4 months vs 1.7 ± 0.7 months; *P* = 0.0161). Higher satisfaction levels were reported among owners of indoor queens than among those with outdoor queens (88.9%, 8/9 vs 38.5%, 5/13, respectively; *P* = 0.0306). Furthermore, owner satisfaction was significantly higher among owners whose cats did not experience reproductive events during the 1-year study period (100%, 11/11) compared with owners whose queens exhibited signs of estrus or became pregnant (18.2%, 2/11; *P* = 0.0001).

## Discussion

Our study aimed to investigate the safety, immunogenicity, and efficacy of Gonacon–rabies vaccination in owner-owned pet queens residing in their natural habitats under their owners’ care. In addition, although a small Ovx–Rabies group was included as a reference, our study focused on differences in vaccination responses and reproductive outcomes between indoor and outdoor queens in the Gonacon–Rabies group. As expected, queens in the Ovx–Rabies group were permanently sterilized and developed protective levels of anti-rabies antibodies. In the Gonacon–Rabies group, the rabies vaccine induced robust immune responses; however, its immunogenicity was inferior in outdoor queens, resulting in significantly lower rabies antibody levels than in indoor queens. We found that Gonacon–Rabies vaccination generated positive anti-GnRH antibody responses in some owner-owned pet queens, which was associated with apparent contraceptive efficacy. However, outdoor queens showed lower immunogenicity to the Gonacon vaccine than indoor queens, and several females experienced reproductive events post-Gonacon vaccination. Owner satisfaction with the Gonacon–Rabies vaccination approach varied by housing conditions and reproductive outcomes, underscoring the need for tailored population control strategies. Overall, our study emphasizes the importance of considering environmental factors and queens’ habitats in rabies vaccine efficacy and immunocontraception efforts for population management.

In our study, the rabies vaccine elicited a robust immune response in all vaccinated queens, with protective antibody levels maintained throughout the study period. This finding is consistent with previous research demonstrating the efficacy of rabies vaccination in cats (Fogelman *et al.* 1993, Mansfield *et al.* 2004, Roebling *et al.* 2014, Shiraishi *et al.* 2014). However, recent reports have documented rabies cases in previously vaccinated dogs and cats, highlighting that vaccination does not always guarantee full protection and emphasizing the importance of monitoring immunity in at-risk populations (Nathan *et al.* 2025). In the current study, outdoor queens exhibited significantly lower rabies antibody concentrations than indoor queens, raising concerns regarding the consistency and durability of vaccine-induced immunity in free-roaming cat populations. Consistent with this finding, we recently reported that outdoor toms vaccinated with Gonacon and rabies exhibited significantly lower rabies antibody levels than indoor toms (Novak *et al.* 2026). Some other previous studies have also identified challenges in achieving and maintaining long-term, sufficient rabies immunity in outdoor cats, suggesting that factors such as vaccine delivery, exposure to wildlife reservoirs, and environmental stressors may play a role in rabies vaccine immunogenicity (Mansfield *et al.* 2004, Roebling *et al.* 2014, Shiraishi *et al.* 2014).

Previous studies using high-dose GnRH antagonists and agonists have shown temporary suppression of reproductive activity in female cats, indicating that modulation of the hypothalamic–pituitary–gonadal axis is a viable contraceptive approach (Goericke-Pesch *et al.* 2014, Velkovska *et al.* 2025). Immunocontraception strategies in domestic cats and other animal species are based on active immunization against key reproductive antigens and on antibody stimulation, which, in turn, neutralize the relevant factor(s) and alter fertility (Kutzler & Wood 2006, Levy *et al.* 2011). Gonacon specifically targets gonadotropin-releasing hormone (GnRH), the master regulator of reproduction. The vaccine contains a GnRH peptide linked to a large carrier protein and formulated with an oil-based adjuvant containing mycobacterial cell wall components, aiming to enhance and prolong antibody production (Benka & Levy 2015, Novak *et al.* 2026). By inducing anti-GnRH antibodies, Gonacon is anticipated to disrupt GnRH signaling, thereby reducing gonadotropin secretion, leading to downstream suppression of folliculogenesis and reproductive activity. In our study, the immunogenicity of the Gonacon vaccine was evaluated by measuring anti-GnRH antibody levels, which varied overall among vaccinated queens and over time. While most cats exhibited initial positive antibody levels, a subset of individuals did not mount a sufficient immune response (a non-responder rate of approximately 32%), or their response was relatively short-lived and declined over time, particularly among outdoor cats. Individual variability in vaccine response has been reported in previous studies by others investigating the immunogenicity of Gonacon in cats housed in well-managed laboratory environments (Levy *et al.* 2004, 2011, Vansandt *et al.* 2017, Fischer *et al.* 2018), and by us in feral queens that were trapped and kept in captivity for a 14-week monitoring period (Novak *et al.* 2021), and in the study we performed in pet toms monitored for 1 year after Gonacon–rabies vaccination (Novak *et al.* 2026). Likewise, Johnson *et al.* (2020) tested a novel phage-based GnRH vaccine in male cats and observed substantial individual variability in immune responses and reproductive suppression, underscoring the difficulty of achieving uniform contraceptive efficacy across different GnRH-based vaccine platforms. However, to our knowledge, the current study represents the first investigation into the combined immunogenicity and efficacy of the Gonacon vaccine with the rabies vaccine in pet queens residing in their natural habitats under the care of their owners, thus reflecting the real-life scenario for a segment of the target population requiring fertility and rabies control. The observed differences in immunogenicity between indoor and outdoor cats suggest that environmental factors may influence individual vaccine efficacy and should be seriously considered in management programs. Stressors associated with outdoor living, such as exposure to pathogens, environmental toxins, and social stress, may impair immune function and compromise vaccine responses (Luzardo *et al.* 2023, Ma *et al.* 2023). This is consistent with the 2020 AAHA/AAFP Feline Vaccination Guidelines, which emphasize that lifestyle factors, including outdoor access, environmental stressors, and overall health status, may significantly influence vaccine-induced immunity and susceptibility to infectious diseases in cats (Stone *et al.* 2020).

The simultaneous Gonacon and rabies vaccines appeared overall safe, with no observed adverse reactions, except for injection–site reactions to the Gonacon vaccine in three queens (none to the rabies vaccine); one indoor and two outdoor queens. This finding is consistent with previous studies reporting mild local reactions following Gonacon administration in some cats (Levy *et al.* 2004, 2011, Vansandt *et al.* 2017, Fischer *et al.* 2018, Novak *et al.* 2026). Interestingly, the three queens with injection-site reactions had consistently high anti-GnRH antibody levels, which persisted for at least 12 months. These three queens also had consistently low serum AMH concentrations, with no reproductive events reported during the study. Together, these observations suggest a possible association between injection-site reactions and increased Gonacon immunogenicity and contraceptive efficacy. However, these injection-site reactions warrant further investigation to elucidate potential contributing factors and to evaluate their clinical significance, management, and implications for vaccine use.

The study demonstrates the ability of the Gonacon vaccine to suppress reproductive activity in vaccinated queens, provided that higher anti-GnRH levels are developed, which is associated with a low incidence of estrus and pregnancy among successfully vaccinated cats. This finding aligns with previous studies (Levy *et al.* 2004, 2011, Vansandt *et al.* 2017, Fischer *et al.* 2018, Novak *et al.* 2021). In addition, queens with positive anti-GnRH levels exhibited reduced serum AMH concentrations, consistent with our previous publication (Novak *et al.* 2021). Our findings further support the value of reduced AMH as a marker for reproductive suppression and the vaccine’s contraceptive effects. As anti-GnRH levels gradually reduced, as illustrated more clearly in outdoor queens, AMH concentrations increased, and reproductive events became more common. This pattern is consistent with findings from queens treated with deslorelin implants, in which AMH concentrations rose as ovarian activity resumed following implant removal (Goericke-Pesch *et al.* 2014, Ferre-Dolcet *et al.* 2022). A comparable finding was also reported in cats treated with Improvac (Zoetis), which is a GnRH vaccine licensed for boar taint prevention in pigs, where estrus and pregnancy returned as the contraceptive effect waned, but a booster dose prolonged suppression in queens (Morgan *et al.* 2019, Florencia *et al.* 2025). The association between anti-GnRH antibody levels and serum AMH concentrations provides valuable insights into the mechanisms underlying the contraceptive effects of Gonacon and the potential biomarkers of ovarian function in cats, particularly since AMH has been reported not to be affected by season in domestic cats (Place *et al.* 2011, Axner & Strom Holst 2015, Claassen *et al.* 2023) and shows clear differences between intact and ovariohysterectomized queens, with additional minor cyclic variation even in nonovulating intact queens (Johnson *et al.* 2023). Moreover, our results highlight the potential of the Gonacon vaccine, but also underscore the need to explore further potentially more effective regimens, such as higher vaccination doses, administration of Gonacon booster vaccinations (as has been shown in other species), more potent immunogenic vaccine formulas, or complementary approaches that would provide more consistent, effective, and longer-lasting strategies to address reproductive control in free-roaming cat populations (Baker *et al.* 2018, Pinkham *et al.* 2022). In addition, future research should also identify alternative biomarkers of ovarian function to improve monitoring of contraceptive efficacy in immunocontracepted individuals.

Contrary to our original hypothesis, indoor and outdoor queens differed substantially in their responsiveness to both anti-GnRH and rabies vaccines. Our study revealed notable differences in immunogenicity, reproductive outcomes, and owner satisfaction. Outdoor queens showed lower immune responses to both the Gonacon and rabies vaccines, experienced more reproductive events, and their owners expressed lower satisfaction than those of indoor queens. Interestingly, in our parallel study of pet toms monitored for 1 year after Gonacon–rabies vaccination, we observed similar indoor–outdoor differences, suggesting a broader, habitat-related effect (Novak *et al.* 2026). Free-roaming outdoor cats may receive less care and attention from their owners than indoor cats and may be exposed to greater social stress (de Assis & Mills 2020, Ma *et al.* 2023). They typically face many challenges, including exposure to harsh weather and noise, as well as risks of injury from other animals, people, and vehicles (Slater 2007, Gerhold & Jessup 2013, Gunther *et al.* 2015, 2018, Crawford *et al.* 2019). Moreover, they are susceptible to infectious diseases and at greater risk of compromised health, resulting in higher morbidity and mortality rates (Slater 2007, Gunther *et al.* 2018, Crawford *et al.* 2019). Indeed, our study found lower PCV in outdoor queens, possibly indicating anemia in some of them. Furthermore, the loss-to-follow-up rate was higher among outdoor queens, highlighting the risks associated with their habitat. Potentially, the suboptimal conditions of outdoor queens may contribute to reduced immune response and antibody production following vaccination with Gonacon and rabies vaccines, as has been suggested for other vaccines and species (Zimmermann & Curtis 2019, Tizard 2021). Our findings highlight key challenges in managing feral and free-roaming cat populations and the associated public health risks, especially given that most free-roaming cats receive only a single lifetime rabies vaccination, typically during TNVR. Strategies aimed at improving the health and welfare of outdoor cats, such as habitat enrichment, proper feeding, and vaccination campaigns, may help mitigate the negative impacts of outdoor living and promote population control in free-roaming cat populations (Levy *et al.* 2003, de Assis & Mills 2020, Lawson *et al.* 2020, Ma *et al.* 2023).

Owner satisfaction with Gonacon–rabies vaccination varied with housing conditions and reproductive outcomes. Indoor queens’ owners reported significantly higher satisfaction levels than outdoor cat owners, likely due to the lower incidence of reproductive events and the perceived efficacy of the Gonacon vaccine in indoor settings. Similarly, owners whose cats did not experience reproductive events were more likely to report greater satisfaction with Gonacon–rabies immunization, underscoring the importance of achieving the desired contraceptive outcome. Notably, owner satisfaction was not influenced by the queens’ immune response to the rabies vaccine, as owners were unaware of these laboratory findings. This highlights a concern, as inadequate rabies responsiveness, if it occurs, may go unnoticed clinically and therefore be overlooked in practice. Future work should also examine how well owners understand the implications of inadequate rabies vaccine responses in free-roaming cats, as these deficiencies may otherwise go unnoticed. Moreover, there is a broader need to strengthen communication not only with owners but also with veterinarians, animal welfare organizations, municipal authorities, policy makers, and other stakeholders, to ensure that expectations and management practices are aligned with the realistic outcomes and constraints of immunocontraceptive and rabies-control strategies (de Assis & Mills 2020, Luzardo *et al.* 2023).

Overall, our study provides new insights into the safety, efficacy, and owner satisfaction associated with combined Gonacon–rabies vaccination in owner-owned queens living in their natural habitats. Our findings demonstrate marked differences in immunogenicity and reproductive outcomes following rabies and Gonacon vaccination between indoor and outdoor queens, underscoring the importance of environmental and housing conditions when implementing reproductive and rabies control strategies. Future research should aim to clarify the mechanisms driving individual variability in vaccine responses and to develop targeted approaches that enhance contraceptive efficacy, particularly in outdoor cats. Overall, this work advances the understanding of feline reproductive health management and supports evidence-based strategies to reduce cat overpopulation while safeguarding public health and animal welfare.

## Supplementary materials



## Declaration of interest

The authors declare that they have no known competing financial or personal interests that could have appeared to influence the work reported in this paper.

## Funding

This work was supported by the Israel Chief Scientist, Ministry of Agriculture and Rural Development (grant number 12-11-0009) and the Hebrew University. The research was also supported in part by the U.S. Department of Agriculture, Animal and Plant Health Inspection Service.

## Author contribution statement

SN contributed to investigation, data curation, visualization, and preparation of the initial draft. BY contributed to conceptualization, resources, validation, and writing (review and editing). RK contributed to conceptualization, methodology, and writing (review and editing). LJ contributed to investigation, formal analysis and writing (review and editing). DCE contributed to conceptualization, resources, validation, and writing (review and editing). TR contributed to supervision, conceptualization, methodology, formal analysis, resources, funding acquisition, visualization, writing of the original draft, and review and editing of the manuscript.
