# Supplementary material for: One-year reproductive effects of anti-GnRH and rabies vaccination in female pet cats
Source: Reprod Fertil. 2026 Oct 19;7(4):RAF260102. doi: 10.1530/RAF-26-0102 (PMC13695436; doi:10.1530/RAF-26-0102)
Supplement: Supplementary file 1 [file RAF-26-0102_supplementary_figure_1.pdf]

# Supplementary Data

## One-year reproductive effects of anti-GnRH and rabies vaccination in female pet cats

Shiri Novak, Boris Yakobson, Roni King, Lauren Jaebker, Douglas C. Eckery, Tal Raz*

***Corresponding author:** Tal Raz, Tal.Raz@mail.huji.ac.il

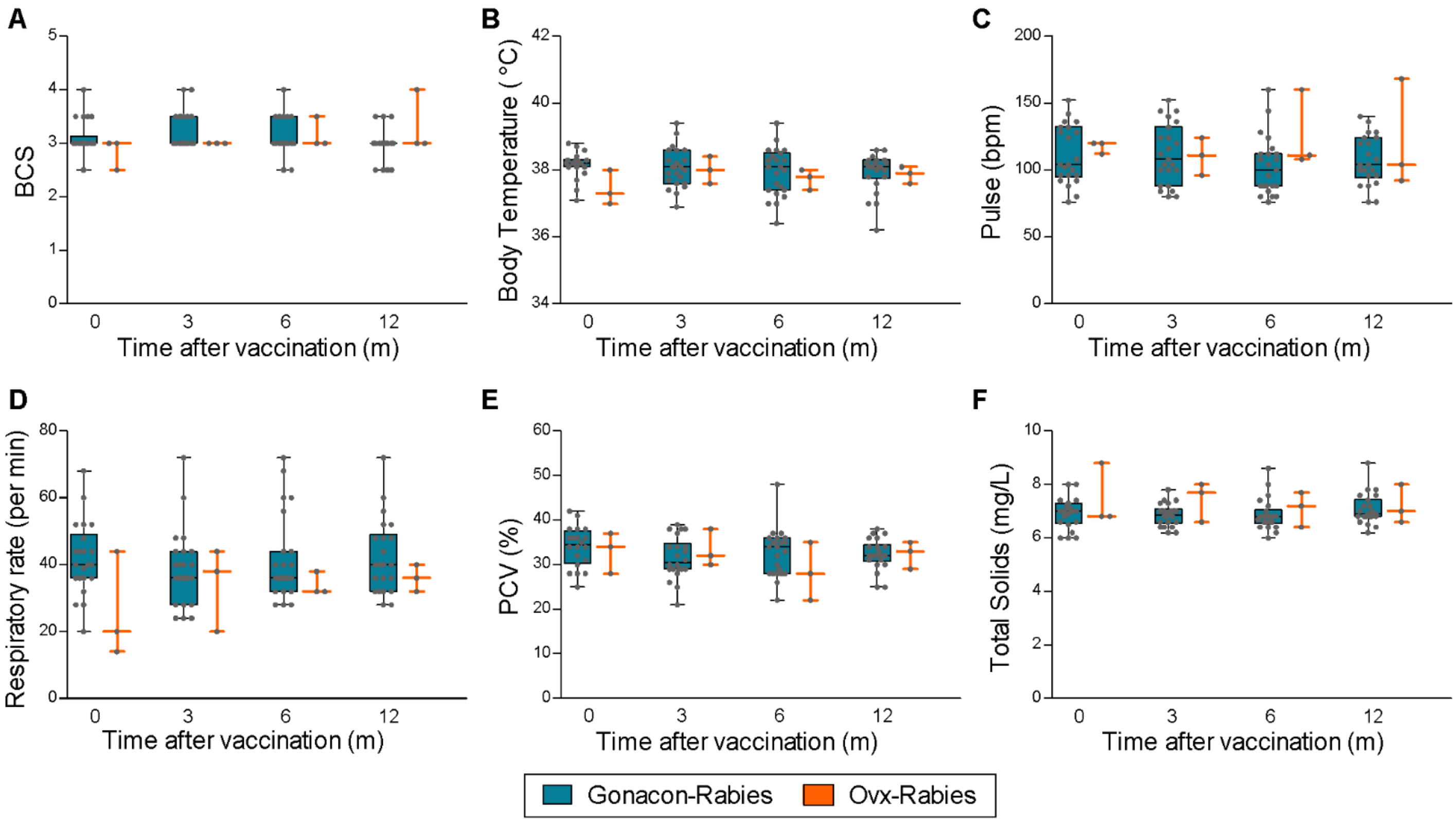


**Supplementary Fig. 1. Body condition score (BCS), vital signs (TPR), packed cell volume (PCV), and total solids (TS) in queens receiving Gonacon-Rabies vaccination or Ovx-Rabies treatment**. Queens in the Gonacon-Rabies group received simultaneous Gonacon and rabies vaccination, whereas queens in the Ovx-Rabies group underwent ovariohysterectomy and rabies vaccination. Measurements were obtained before treatment and at 3, 6, and 12 months thereafter. Boxplots show median values (central line),

interquartile ranges (box), minimum and maximum values (whiskers), with individual observations overlaid (each dot represents a value of a single sample). Data were analyzed using Repeated-Measures ANOVA with Group (between-subject factor), Time (within-subject factor), and Group × Time interaction effects, followed by Tukey HSD pairwise comparisons when appropriate. (**A**) BCS (Group $P$=0.6690, Time $P$=0.0633, Group × Time $P$=0.1631). (**B**) Temperature ($P$=0.8353, $P$=0.0851, $P$=0.4790). (**C**) Pulse ($P$=0.2410, $P$=0.9058, $P$=0.3870). (**D**) Respiratory rate ($P$=0.2338, $P$=0.4159, $P$=0.2548). (**E**) PCV ($P$=0.7139, $P$=0.4749, $P$=0.4676). (**F**) TS ($P$=0.2121, $P$=0.5658, $P$=0.2991).
